# Does Plant Species Richness Guarantee the Resilience of Local Medical Systems? A Perspective from Utilitarian Redundancy

**DOI:** 10.1371/journal.pone.0119826

**Published:** 2015-03-20

**Authors:** Flávia Rosa Santoro, Washington Soares Ferreira Júnior, Thiago Antônio de Souza Araújo, Ana Haydée Ladio, Ulysses Paulino Albuquerque

**Affiliations:** 1 Laboratory of Applied and Theoretical Ethnobiology, Department of Biology, Federal Rural University of Pernambuco, Dois Irmãos, Recife, Pernambuco, Brazil; 2 Ecotono Laboratory, Universidad Nacional del Comahue, CONICET, Quintral 1250, Bariloche, Río Negro, Argentina; College of Medicine, University of Ibadan, University College Hospital, Ibadan, Nigeria

## Abstract

Resilience is related to the ability of a system to adjust to disturbances. The Utilitarian Redundancy Model has emerged as a tool for investigating the resilience of local medical systems. The model determines the use of species richness for the same therapeutic function as a facilitator of the maintenance of these systems. However, predictions generated from this model have not yet been tested, and a lack of variables exists for deeper analyses of resilience. This study aims to address gaps in the Utilitarian Redundancy Model and to investigate the resilience of two medical systems in the Brazilian semi-arid zone. As a local illness is not always perceived in the same way that biomedicine recognizes, the term “therapeutic targets” is used for perceived illnesses. Semi-structured interviews with local experts were conducted using the free-listing technique to collect data on known medicinal plants, usage preferences, use of redundant species, characteristics of therapeutic targets, and the perceived severity for each target. Additionally, participatory workshops were conducted to determine the frequency of targets. The medical systems showed high species richness but low levels of species redundancy. However, if redundancy was present, it was the primary factor responsible for the maintenance of system functions. Species richness was positively associated with therapeutic target frequencies and negatively related to target severity. Moreover, information about redundant species seems to be largely idiosyncratic; this finding raises questions about the importance of redundancy for resilience. We stress the Utilitarian Redundancy Model as an interesting tool to be used in studies of resilience, but we emphasize that it must consider the distribution of redundancy in terms of the treatment of important illnesses and the sharing of information. This study has identified aspects of the higher and lower vulnerabilities of medical systems, adding variables that should be considered along with richness and redundancy.

## Introduction

Resilience can be understood as the ability of a system (whether individual, social, ecological or social-ecological) to restructure itself after the occurrence of a disturbance and to absorb changes without losing the functions, relationships, identity and processes that maintain the system [1,2,3]. Because it involves several variables and interactions between variables, resilience is very difficult to measure, as noted by Ungar [4]; however, we emphasize that select tools can help in understanding certain aspects of system resilience and that this understanding is crucial for determining the permanence of a system in a given regime. In this sense, we focus on the importance of studying Local Medical Systems’ resilience, considering its relevance to local communities that have limited access to public health care services and to the permanence of traditional ecological knowledge.

Local Medical Systems (LMSs) can be defined as complex systems in which protective measures against injury involve knowledge, behaviors and beliefs surrounding health, illness, medicinal natural resources and social actors in small populations, such as rural and indigenous communities [5,6]. Thus, the resilience of an LMS can be observed when, following a disturbance, the system does not abandon its essential processes and functions, such as those involving disease diagnosis, expert recognition and treatment [3]. For example, if local medicinal practices are based on plants, Western medicine may be adopted if particular species become unavailable for treating certain disorders (see intermedicality in [7]), preserving the functions of the system. However, if this change in strategy leads to a transformation of processes that govern the system, as in the instances described above, a transition to another regime will reveal vulnerability in the system, challenging its resilience.

To investigate the resilience of LMSs, Albuquerque & Oliveira [8] introduced the Utilitarian Redundancy Model (URM). This model was based on ecological redundancy [9], which draws attention to the functional aspect of biodiversity. According to Walker [9], functional diversity and ecological redundancy are more important than species diversity and are essential for ensuring the long-term persistence of an ecosystem. Similarly, the URM predicts that a larger number of medicinal species used for the same therapeutic function (species redundancy for the treatment of a specific illness) contributes to the maintenance of a given function. This prediction is based on the ability of one or various species to perform a particular function if an event compromises the use of one particular species that executes that function. Therefore, from a broader perspective, higher numbers of redundant functions in an LMS will correlate with higher degrees of system flexibility and, consequently, of system resilience from a functional perspective.

According to the model, certain species may be preferred and, therefore, may be used more often than others with the same function. Thus, the non-preferred species serve as “stock knowledge” [10] and are essential for resilience when disturbances compromise access to the preferred species. Questioning this prediction, Ferreira Júnior et al. [11] observed that redundant species are not always used in the absence of preferred species because people can search for other strategies, such as using biomedical resources or other practices beyond the LMS. Despite this observation, those authors did not assess the degree to which other strategies are chosen in preference to redundant species.

Several intrinsic aspects of the LMS process should be considered when applying the URM. For example, Ladio and Lozada [12] have noted that investing greater amounts of medicinal resources in the most important categories will increase the adaptiveness and resilience of an LMS because the need for healing directs cognitive innovations, which in turn results in the prioritization of diseases that are more important for the population. In this context, we emphasize the importance of focusing on the role of redundancy in the treatment of illnesses that are more frequent and more severe. This approach facilitates the verification of redundancy in terms of important diseases.

Another factor to be considered is the extent to which redundancy is shared. Even if redundancy is high, the resilience of an LMS can be compromised if the knowledge is restricted to one person, as he or she can abandon the system without disseminating the knowledge. Alternatively, high levels of redundancy may reflect cases in which knowledge is non-uniformly distributed among different individuals so that each individual possesses knowledge of the use of a different species in relationship to the treatment of illness. Although this scenario is less harmful to LMSs than the first, an ideal condition for resilience would involve a situation in which knowledge of most diseases and their treatments is shared [13].

Based on these considerations, this study aims to address several gaps in the URM concept that have not been examined in previous studies but that are essential to the proper use of the model. This study also strives to assess high- and low-vulnerability features of two plant-based medical systems in the Brazilian semi-arid zone. In view of the features that contribute to LMS resilience, the following hypotheses were formulated: 1. There is a predominance of redundant functions in LMSs. 2. The use of redundant species represents the main treatment strategy if preferred species are not accessible. 3. Redundancy is associated with the frequency of illnesses. 4. Redundancy is associated with the severity of illnesses. 5. The knowledge of treatments of illness is shared among local experts.

## Materials and Methods

### Study area

The study was conducted in two rural communities in the municipality of Crato, Ceará state, northeastern Brazil: Sítio Bréa [“Bréa Ranch”] (community center located at S07°05’53.04,” W039°31’23.0”) and Assentamento 10 de Abril [“April 10 Settlement”] (community center located at S07°04’29.7,” W039°28’44.1”). These communities were selected based on prior information about their intensive use of medicinal plants. Subsistence farming represents the major economic activity for both communities, and the cultivation of home gardens, especially for medicinal use, is very common.

The communities are located in the Cariri micro-region [14], an area with deciduous, thorny and succulent species of xerophytic vegetation characteristic of the Caatinga environment [15]. The climate is characterized as mild, hot, tropical and semi-arid, with marked features of seasonality and a rainy season between January and May [16]. These typical Caatinga seasonality features result in major water deficits throughout the year, severely limiting the volume of available water [15].

According to data from health agents serving the region, the Sítio Brea community is composed of approximately 100 families. Although no records have been maintained from the early establishment period, locals estimate that the first houses were built on the site during the 1940s and 1950s. The community is located 24 km from the center of Crato, and access to urbanized areas is facilitated by the CE-55 highway. There are no clinics in the community, and public health services are provided by a health agent who primarily monitors the health of children of up to two years of age as well as elderly residents with chronic diseases such as diabetes and hypertension. Sick individuals rarely seek treatment from this agent and typically resort to plant remedies or, in a lower proportion, industrial pharmaceuticals or care services offered through health centers in the city of Crato.

The Assentamento 10 de Abril community is composed of 47 families that have resided in the community since the settlement’s formation in 1991. The settlement was established as a result of the Landless Workers' Movement (Movimento Sem-Terra—MST) and, although relatively new, is closely linked to the history of the locality. The settlement is located 31 km from the center of Crato, but residents rarely visit the health centers of the city due to the poor condition of the dirt road that connects the community to highway CE-55. The health agent who operated in the settlement had developed a collective project for the cultivation of medicinal plants called “Farmácia Viva” (Living Pharmacy). However, she had moved away from the settlement in the year prior to the beginning of this study. This change resulted in an abandonment of the project by the population, and the community is currently without health care from the government. Despite the abandonment of “Farmácia Viva,” the knowledge disseminated by this health agent has proven valuable to the residents because many of them attribute their knowledge of the use of various medicinal plants to her teachings.

### Ethics statement

Written informed consent was obtained from each participant involved in this study. Information provided by all participants was kept confidential and was anonymously tracked using an identification number. The study was submitted to the Board of Ethics in Research of Pernambuco University and was authorized under the number 351.068, Certificate of Presentation for Ethical Appreciation (Certificado de Apresentação para Apreciação Ética—CAAE) No. 01578012.5.0000.5207.

### Data collection

Data collection was performed during seven visits between March 2012 and May 2013. The visits lasted from one week to one month. During this period, it was possible to participate in various daily community activities to collect additional data through direct observation.

All information obtained throughout the study, including concepts surrounding disease and the frequencies and severities of certain diseases, was obtained based on the perceptions of local experts, who are defined as individuals recognized by a community as possessing most of the knowledge of a medical system [6]; all considerations are therefore limited to this component of the LMS. As expert perceptions of what constitutes an ill state do not always correspond with Western biomedical conceptions of disease, the term “therapeutic target” was used in place of the term “disease”. Therapeutic targets can refer to symptoms such as fever and coughing or to a set of symptoms that constitute a more complex condition such as influenza.

In selecting local experts, intentional, non-probabilistic sampling was performed using the snowball technique [17]. The representatives of the resident associations of the two communities were accessed, as these individuals exhibited considerable knowledge of all the residents. These representatives were asked to identify individuals whom they considered to be local experts in medicinal plant use. This question was posed to each representative until no new individuals were mentioned. As a result, 21 experts were selected for Sítio Brea (18 women and three men, from 36 to 80 years of age) and 25 were selected for Assentamento 10 de Abril (14 women and 11 men, from 23 to 68 years of age).

**Fig 1:**
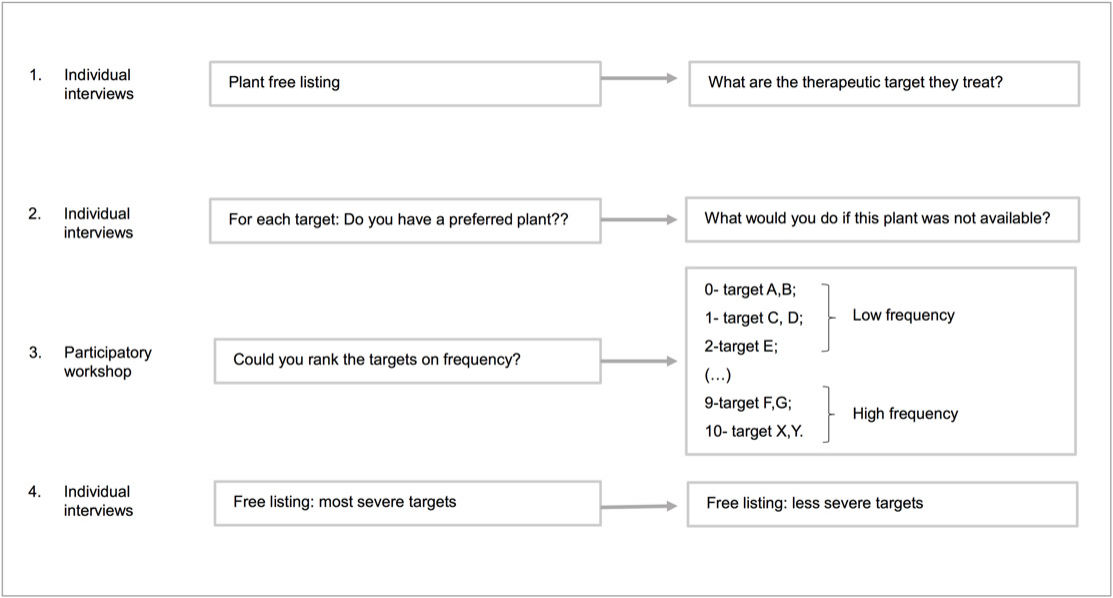
Data collection steps.

Data collection was conducted over four stages, three of which consisted of individual semi-structured interviews and one of which involved a participatory workshop (Fig. 1).

Individuals were first surveyed about medicinal plants that they were aware of and about therapeutic targets that have been treated. For this purpose, the free-listing technique [18] was used, in which informants were asked to name all known medicinal species. Based on this list, the interview script included questions on modes of use, therapeutic targets that are treated and their characteristics.

To address the second hypothesis, it was assessed whether participants held a preference for a certain plant for treating therapeutic targets cited as being treatable with more than one plant (i.e., with redundancy). Thus, the informants were reminded of the species cited for each therapeutic target, and it was asked whether they preferred certain species among the redundant ones. When positive responses were given, a hypothetical situation was presented in which the preferred species was not available, and the participant was asked about what he or she would do. The intention behind this question was to determine whether the main treatment strategy actually involved the selection of redundant species or whether other strategies were used more frequently.

The frequency of therapeutic targets, the focus of the third hypothesis, was estimated through a participatory workshop in which the consensus of the informants was considered [19]. Although all the experts from both communities were invited to participate in the workshop, only 19 (90%) attended the workshop held in Sítio Bréa, while 21 (84%) attended the Assentamento 10 de Abril workshop. Throughout the activity, cards with the names of therapeutic targets cited during the interviews were distributed, and the experts were asked to attribute a value ranging between 0 and 10 to each target based on the frequency at which they appeared. A score of 10 corresponded to the most frequent targets, and a score of 0 corresponded to those that never occurred.

In assessing the perceived severity of a therapeutic target, individual semi-structured interviews were conducted, again using the free-listing technique, in which informants were asked to first list all therapeutic targets they deemed most severe and, then, to list those targets that they considered to be the least severe. Experts occasionally cited therapeutic targets that had not been previously mentioned or that could not be treated with plants. As this study focuses on the use of medicinal plants, such targets were not included in the analysis, but they will be presented as a component of local knowledge of existing illnesses.

Visits to home gardens and guided tours through native forests [18] were conducted in each community to collect and identify plants by their traditional names. To confirm the identity of the local plant taxa, at least two informants were consulted following the collection of each plant [18]. Species were identified with the assistance of taxonomists and were then stored at the Herbarium Caririense Dárdano de Andrade-Lima of the Regional University of Cariri (Universidade Regional do Cariri).

### Data analysis

Therapeutic targets were identified in accordance with local nomenclature; however, the described symptoms of these targets were closely analyzed, and these data were classified by affected body systems according to classification systems provided by the World Health Organization [20]. A number of therapeutic targets cited by different informants with distinct names presented very similar descriptions. In these cases, informants were asked whether the differing names cited would correspond to a single target. If the answer was positive, the most frequently cited name was adopted.

Thus, all therapeutic targets were grouped into three levels of redundancy, according to the classification scheme provided by Albuquerque and Oliveira [8]: “highly redundant,” targets with responses that included more than 15% of all plant species; “redundant,” targets whose number of species listed for treatment represented between 5% and 15% of the species cited; and “less redundant,” targets whose species represented less than 5% of all species. In this study, we considered it important to examine targets exhibiting no indications of redundancy, i.e., in which only one single taxon could be used for treatment, as well as targets that the LMS could not treat. Therefore, within the “less redundant” category, “non-redundant” targets were distinguished from “without treatment” targets. The latter were not included in the data analysis because they were not treated through the plant-based LMSs.

The hypotheses and tests used to answer the research questions are presented in Table 1. To address the first hypothesis, the proportion of the targets classified as redundant and highly redundant was compared with the proportion of the targets classified as less redundant using a Chi-Squared test (one sample). For the second hypothesis, the same test was used, comparing the proportions of strategies mentioned when the informants were presented with the hypothetical scenario involving the absence of a preferred species. As each informant was asked about alternative strategies for each target in the absence of preferred species, the number of citations of strategies exceeds the number of informants and targets.

**Table 1:** Hypotheses and predictions tested in this study and statistical tests employed.

Hypothesis	Prediction	Test
**There is a predominance of redundant functions in the LMSs.**	It is expected that the proportion of redundant and highly redundant therapeutic targets is greater than the proportion of less redundant targets.	χ^2^(one sample)
**The use of redundant species is the main strategy when preferred species are not available.**	It is expected that the proportion of responses indicating the use of redundant species is greater than the proportion of responses indicating any other strategy.	χ^2^(one sample)
**Redundancy is associated with the severity of the therapeutic target.**	It is expected that the average number of species known to treat severe therapeutic targets is greater than the average number of species known to treat non-severe targets.	Mann-Whitney test
**Redundancy is associated with the frequency of the therapeutic target.**	It is expected that the number of species known to treat a therapeutic target is associated with the frequency rank assigned to this target.	Spearman correlation
**Knowledge of the treatment of therapeutic targets is shared among local experts.**	It is expected that the majority of therapeutic targets and information units are familiar to approximately one-half of the experts.	Normality test: Lilliefors

For the third hypothesis, the Spearman correlation coefficient was used to correlate the rank of each therapeutic target (from the participatory workshop) with the number of taxa with which each one could be treated. Regarding the fourth hypothesis, taxon quantities associated with severe targets were compared with taxon quantities associated with non-severe targets using a Mann-Whitney test.

The fifth hypothesis was analyzed from two perspectives. First, the analysis aimed to assess whether experts were aware of treatments for the same therapeutic targets. In other words, although knowledge sharing about the targets may occur, this knowledge does not necessarily concern the medicinal plants that are used to treat them. It also aimed to assess whether complete information on therapeutic targets and medicinal plants used to treat them was shared between experts. For this purpose, data were separated into “therapeutic target—medicinal plant” information units, as proposed by Winter and MacClathey [21]. For example, if the “cold” therapeutic target could be treated by two redundant medicinal plants, such as mint and eucalyptus, two units would be related to cold treatment: “cold-mint” and “cold-eucalyptus.” Because the analyses are limited to responses from experts, it is assumed that degrees of sharing could be evaluated based on a situation in which most of the available information units are known by approximately half of the experts. This situation can be seen when the data follow a normal distribution (see [13]); therefore, the Lilliefors test was used. All statistical tests were performed with BIOESTAT 5.0 [22] software.

## Results

### The local plant-based medical system

Experts from both communities cited a total of 157 medicinal plants, of which 145 taxa could be identified, belonging to 60 families. Two of these taxa are deemed endangered according to the IUCN [23]. Certain species were only identified at the genus level, and one species was only identified at the family level. In addition, specimens were not found for 12 local taxa, and these taxa were, therefore, not properly identified by their scientific names. The richness of known medicinal species was found to be 126 for Sítio Bréa, of which 59 were identified as preferred, and 125 for Assentamento 10 de Abril, of which 43 species were preferred for certain treatments.

Experts recognized 90 therapeutic targets in each community, 18 of which cannot be treated with medicinal plants in Sítio Bréa and 12 of which cannot be treated with medicinal plants in Assentamento 10 de Abril (S1 Table). According to the experts, these illnesses were usually treated with the aid of commercial drugs or in hospitals. Furthermore, the use of commercial drugs was occasionally observed in combination with the use of medicinal plants for illnesses treated by the LMSs.

The most frequently mentioned therapeutic targets corresponded to disorders of the digestive system (16 targets in Ass. 10 de Abril and 13 in Sítio Bréa) and respiratory system (13 targets in Ass. 10 de Abril and 10 in Sítio Bréa) and to infectious/parasitic diseases (nine targets in Ass. 10 de Abril and seven in Sítio Bréa). Although a number of these targets may be considered the same disease according to a classification based on Western medicine, for some targets, it was not possible to establish an equivalent. S2 Table shows the equivalent condition of Western medicine and a detailed explanation of those targets when it was not possible to establish equivalence. All data from this study are stored in the Laboratory of Applied and Theoretical Ethnobiology database and are available for review with the signing of a confidentiality agreement and an agreement that protects those involved in the study.

### Redundancy in the Local Medical Systems

Contrary to what was expected, a predominance of less-redundant therapeutic targets was found in the LMSs studied (S1 Table), corresponding to 69% of the therapeutic targets identified in Sítio Bréa and 77% in Assentamento 10 de Abril. These proportions are significantly higher than those of the redundant and highly redundant targets (χ^2^ = 11.306 and χ^2^ = 13.764, respectively, with p<0.005). Among these targets, several can only be treated by a single taxon and are thus labeled under the “no redundancy” category (20% for Sítio Bréa and 18% for Ass. 10 de Abril).

### The use of redundant species

**Fig 2:**
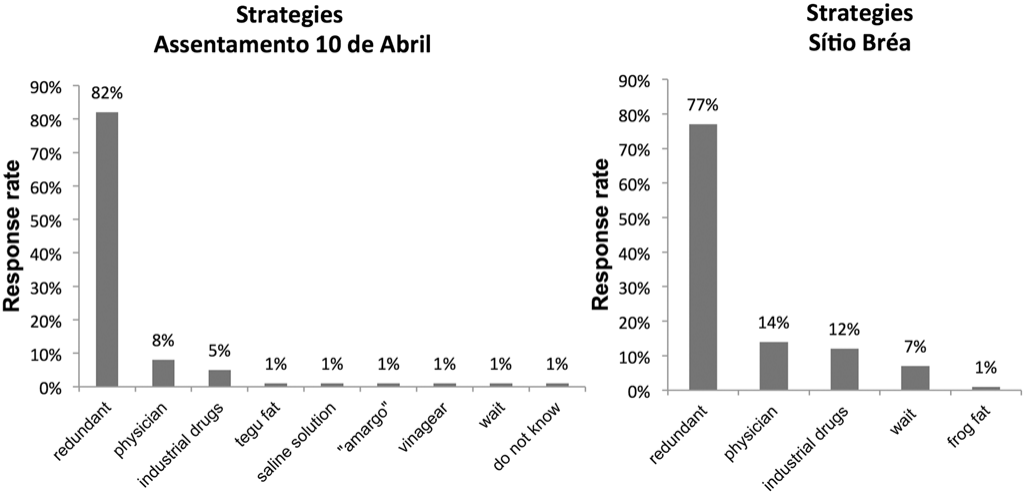
Citation rates for local experts on treatment strategies in the absence of a preferred species in the medical systems of two rural communities in the semi-arid region of northeastern Brazil.

When the absence of a preferred species was simulated, the proportion of responses that involved the use of other available species (redundant for the same function of the preferred species) was significantly higher than the proportion of responses referring to the use of any other treatment in Sítio Bréa (χ^2^ = 43.45, p<0.0001) and in Assentamento 10 de Abril (χ^2^ = 64.9 3, p < 0.0001) (Fig. 2). Thus, the substitution of medicinal plants similar to preferred ones was the dominant response, as predicted by the model.

On several occasions, despite being aware of other taxa that serve the same function as preferred plants, experts cited other treatments, such as resorting to physicians, the use of commercial drugs and, to a lesser extent, the use of medicinal animals and other strategies, such as the use of “amargo” (a commercial phytotherapic composed of *Baccharis genistelloides* L., *Matricaria chamomilla* L., *Camellia sinensis* (L.) Kuntze and *Mentha piperita* L.), vinegar and saline (Fig. 2). Informants occasionally (less than 1%) cited these strategies, even for cases when the preferred species was available. On rare occasions, experts could not determine what they would do without access to the preferred species or indicated that they would simply wait for the therapeutic target to heal before using other taxa known to provide similar functions.

### The frequency and the severity of therapeutic targets and the relationship with the redundancy of plants

A correlation was found between the frequency of occurrence of the therapeutic target and the redundancy of medicinal plants used to treat it. The Spearman correlation coefficient was found to be rs = 0.5904 (p<0.001) for Sítio Bréa, whereas for Assentamento 10 de Abril, it was calculated to be rs = 0.4036 (p<0.001). This result shows that in both communities, the frequency of a therapeutic target was associated with the number of taxa that were known to treat it.

When redundancy was related to perceived severity, the results showed an interesting trend. According to a Mann-Whitney test (p<0.05), the therapeutic targets considered to be non-severe showed higher levels of medicinal plant redundancy ($\overset{-}{x}$ = 10.26±10.39 in S. Bréa and $\overset{-}{x}$ = 10.73±8.05 in Ass. 10 de Abril) than the most severe targets ($\overset{-}{x}$ = 3.35± 4.86 in S. Bréa and $\overset{-}{x}$ = 5.57±6.72 in Ass. 10 de Abril). Therefore, contrary to what was expected, the LMSs were structured by aggregating a smaller number of taxa to treat more severe targets. S1 Table shows the therapeutic targets listed as severe and non-severe in the scaled lists and their respective medicinal plant richness values.

These results illustrate that low-severity is inversely related to high-frequency. S1 Table shows that therapeutic targets listed as non-severe represented a higher proportion of rankings of five and higher provided through the participatory workshop with the two communities (72% and 83%), and the most severe targets represented a higher proportion of rankings ranging between 0 and five for Sítio Bréa (70%) but represented a lower proportion of targets in the case of Ass. 10 de Abril (25%).

### Sharing the knowledge of treatments

**Fig 3:**
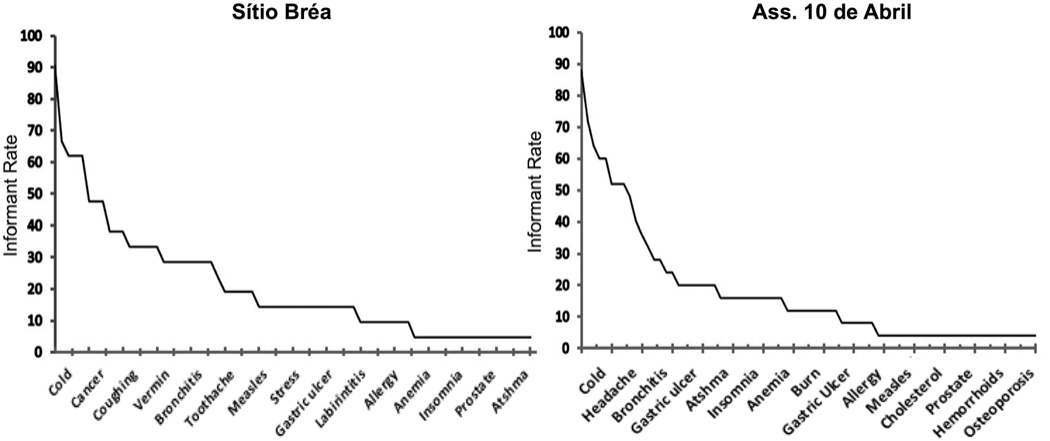
Rate of sharing of therapeutic targets among local experts from two rural communities in the semi-arid region of northeastern Brazil.

In analyzing the proportion of informants citing the same therapeutic targets (independent of sharing information on the taxa used), it can be verified that knowledge was not evenly distributed among the local experts. In Sítio Bréa, knowledge of treatments for 26% of the therapeutic targets was exclusive to certain informants (S1 Table); i.e., if one of these targets occurred, only one expert would know how to treat it (Fig. 3). The most frequently cited therapeutic targets were mentioned by 90% (cold), 67% (uterine inflammation) and 62% (headache, fever and “food that offends”) of the experts; the other targets were cited by less than one-half of the experts (Fig. 3). For Ass. 10 de Abril, 35% of the knowledge of therapeutic target treatments is exclusive (S1 Table), and the most cited targets were mentioned by 88% (cold), 72% (cut), 64% (fever), 60% (blow and “food that offends”) and 52% (uterine inflammation, stomach ache and headache) of the experts. The mean number of citations for each therapeutic target was very low in both communities, reaching 4.36±3.77 in Sítio Bréa and 4.28±4.50 in Ass. 10 de Abril. The results of a Lilliefors test reinforced these results, demonstrating that the distribution of experts who shared information on each therapeutic target did not fit a normal distribution (p < 0.01) in either community.

**Fig 4:**
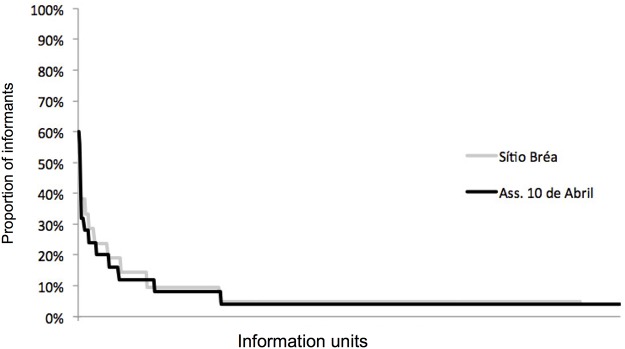
Rate of sharing of information units (therapeutic target—medicinal plant) among local experts from two rural communities in the semi-arid region of northeastern Brazil.

In addressing “therapeutic target—medicinal plant” information units, i.e., the analysis that considers the effect of sharing complete information on target treatment, the number of information units was found to be 421 for Sítio Bréa and 483 for Assentamento 10 de Abril. Among these, 306 (73%) and 356 (74%), respectively, were single citations, i.e., unshared information (Fig. 4). In Sítio Bréa, the most frequently shared units were cited by 52% (“cold-malva do reino”) and 38% (“cold-hortelã”, “headache-hortelã”, “wound-aroeira”, “uterine inflammation-malva do reino” and “blow-mentruz”) of the experts. In Assentamento 10 de Abril, the most frequently cited information units were shared by 60% (“cold-malva do reino”) and 56% (“cut-aroeira”) of the experts. The mean unit sharing value was 1.63±1.30 in Sítio Bréa and 1.62±1.50 in Ass. 10 de Abril. Considering the number of experts in each community (21 in Sítio Bréa and 25 in Assentamento 10 de Abril), this mean number represents a low level of information sharing. The results of a Lilliefors test showed that the distribution of the number of experts who cited each information unit was not normal (p<0.01) in either community.

If both analyses are considered, it is evident that although there is little sharing according to both perspectives, knowledge of medicinal plant treatments is more widespread than treatment information overall when both the treatment and resource are examined. For example, in the case of idiosyncratic information, the proportion of non-shared information units (73% and 74%) was more than twice the proportion of non-shared therapeutic targets (26% and 35%). Moreover, the mean sharing value of information units was found to be more than twice the mean sharing value of therapeutic targets. This difference shows that even if a target is widely known, experts do not necessarily treat a target with the same taxa.

## Discussion

### Redundancy in the Local Medical Systems

The LMSs of the two examined communities are remarkably rich, compared to communities examined in the same region by other studies [8, 24]. Based on these data, an analysis of resilience that considered only species richness would indicate that such LMSs are resilient. However, as low degrees of functional analogy were identified, the richness of species may not reflect actual protection against disorders.

Although several authors have recognized the effect of redundancy on the resilience of socio-ecological systems [25,26,27], to our knowledge, there are few studies that can serve as a comparison. Brown et al. [28] used a different but very interesting approach to utilitarian redundancy. They sought to verify the diversity of useful species in forest fragments and considered that fragments that possess several species with the same utilitarian function contribute to maintaining this function in local communities. This approach is fundamental to check the availability of wild species in preserving utilitarian functions, although it does not consider access to cultivated species. As this work did not use the same perspective, it cannot serve as a comparison.

Among the studies that used the URM, the one conducted by Albuquerque and Oliveira [8] generated results similar to ours, while works by Ferreira Júnior et al. [11] and Ferreira et al. [29] reported a predominance of redundant categories in examined medical systems. These latter studies, however, analyzed LMSs from perspectives that differ from the former, which may have influenced the results. Ferreira Júnior et al. [11] studied only therapeutic targets falling under the category “inflammation”, whereas Ferreira et al. [29] investigated the use of medicinal animals in street markets, where information can freely circulate between different LMSs.

According to the URM, the low degree of functional analogy in an LMS, such as that found in the communities of the present study, results in high degrees of vulnerability [8] due to limited levels of flexibility in the treatment of an illness. The Caatinga vegetation has marked seasonality, and certain medicinal herbs are not available year-round [15]; consequently, in the event of an illness, people will not have access to many alternatives, especially in the case of illnesses that can be treated with only a single species. This situation can occur in any environment where some plant parts may not be available in some seasons.

More important than the unavailability of species due to seasonality are cases of a local extinction of species. In seasonal environments, which have cycles, it is expected that communities will have strategies to cope with these cycles. For example, in Caatinga, they can use tree bark, which is available throughout the year, instead of herbs (see [10]). However, local extinctions of species are somewhat unpredictable, and the lack of redundant species to sustain the LMS in this situation may represent a risk to its permanence. Local extinction can be caused by deforestation, such as that generated by accidental fires, or by overexploitation. In this context, Stewart [30] reported that the extraction of the bark of a widely used medicinal species has led to a high mortality of reproductive individuals and to a decrease in fruit production and seedling survival, indicating that the persistence of the species in the region is threatened.

Another decisive factor is local extinction of medicinal knowledge. For example, if a local expert leaves the LMS for some reason, such as migration or death, some exclusive knowledge can disappear with him or her if it has not been shared. Thus, we underscore the importance of the redundant species to support the LMS in case of a disturbance such as those cited above and emphasize that analyzing species redundancy, apart from species richness, is essential to investigate the persistence of a system based on natural resources.

### The use of redundant species

The URM prediction that people will use other available plants in the absence of preferred ones was confirmed by local experts, demonstrating an aspect of low vulnerability in this medical system. If there is preference for a certain plant, this plant acquires a role in the LMS that is not performed by other plants [10]. In this case, preferred species are similar to the “driver species” described by Walker et al. [31] in their work on ecological redundancy. According to these authors, such species are the main species responsible for ecosystem functioning, and other species that perform the same function but on a smaller scale can be called “passenger species”. Walker et al. [31] stated that, depending on the environmental conditions, “passenger species” may assume the role of “driver species”, ensuring the survival of ecosystem functioning.

When a hypothetical disturbance that would lead to the disappearance of preferred plants was proposed, experts predominantly stated that they would resort to using another plant species. In this scenario, the alternative species would assume the role of the preferred species, and the therapeutic function would still be fulfilled using plant species. As stated above, this can be crucial in cases such as local extinction.

Commercial drug use and physician visits were occasionally cited as alternative solutions to the hypothetical situation presented; however, it cannot be concluded that the presence of biomedicine is harmful to LMS resilience. These alternatives may instead be part of the LMS, thus supplementing the system’s resilience. In addition, other factors, aside from the use of medicinal resources, may interfere with system resilience, such as local beliefs and the reputations of experts. However, this study only examined the medicinal plant treatment aspects of the LMS because plants have previously been shown to be the most significant medicinal resource to the examined communities. Although the degree to which the adoption of Western medicine and other elements may interfere with the local medical systems is not known, it seems reasonable to consider, based on present data, that the redundancy level of medicinal plants is the major factor responsible for maintaining therapeutic functions and, thus, for maintaining LMS resilience.

URM predictions on the use of redundant species have not been previously tested. This study is the first to test these predictions as hypotheses, and such a task has been discussed in previous studies [11,32]. In generating broader acceptance of these URM predictions, future studies may assess all the strategies applied by LMSs when faced with real disturbances and may test this hypothesis across different socio-ecological systems.

### The frequency and the severity of therapeutic targets and the relationship with the redundancy of plants

In examining factors related to expert perceptions of therapeutic target characteristics, it was found that the selection of medicinal plants was inversely related to the severity of the illness, as the majority of therapeutic targets that were cited as most severe were less redundant. This result may lead to three interpretations:

1) Experts may aggregate fewer taxa for the treatment of severe therapeutic targets due to risks associated with experimenting with alternative species when there is a high risk of death, which is not the case when the illness is not considered severe. Fears of experimenting with alternative species in such cases may be disadvantageous to LMS resilience because they reduce the redundancy levels of severe therapeutic targets. However, this approach may be essential to the resilience of the sick individual (see individual resilience in [4]). According to Laland [33], as the production of new knowledge requires high levels of energy expenditure, it is more advantageous to follow pre-existing information in dangerous situations, such as in cases of severe illness. Even if experimentation is rare in LMSs, considering the time at which the communities were established, it is probable that some experiments have occurred, favoring less severe therapeutic targets. Laland [33] also states that knowledge generation is most common in unstable environments, where individuals are presented with new challenges and thus cannot refer to pre-existing knowledge. This explanation may apply to communities of Caatinga, where significant degrees of climatic instability have occurred over recent years, resulting at times in unpredictable dry- and rainy-season weather patterns [15]. Another situation in which innovation could favor redundancy can be found in nomadic communities, which are constantly facing new variable environments (see [34]). Environmental instability may result in the unavailability of certain species even in seasons when they used to be available, leading experts to search for new alternatives, e.g., seeking exotic species in the city. This instability can also promote the emergence of new diseases, which can motivate people to try new species.

2) Processes of medicinal plant selection can change over time due to flaws in knowledge transmission [35,36]. Surplus information in the system may therefore result from “maladaptive” mutations (see [37]), i.e., species that may not serve as a treatment but that are included in the system due to inefficiencies in the diffusion of knowledge, which may promote an increase in treatment redundancy. These failures are more likely to occur in the case of non-severe therapeutic targets, which are less likely to cause major damage to the individual than severe targets that can lead to major complications and that may be more hastily excluded from the system, resulting in higher levels of non-severe therapeutic target redundancy.

3) More severe therapeutic targets may also be more complex and may thus need to be treated with chemical compounds that are exclusive to certain taxa. Less complex targets may, conversely, be treated by a larger set of distinct chemical compounds found in many taxa (see [38]).

A fourth factor that may explain these results is the frequency with which severe illnesses occur. We observed that severe therapeutic targets are substantially less frequent in Sítio Bréa and that those considered non-severe are among the most frequent in both communities. Experts may thus be focusing their efforts on learning and storing knowledge about events that they consider to be real and recurring.

Confirming this hypothesis, experts showed higher levels of knowledge of medicinal plants that treat the most frequent therapeutic targets than of medicinal plants that treat the least frequent targets. From an adaptive point of view, concentrating efforts on the acquisition of information related to frequent events is advantageous, and thus, this aspect confers resilience to the system [12]. Experts are more likely to collect information on species that treat more prevalent therapeutic targets through cognitive methods such as trial and error.

In addition, cultural knowledge, such as knowledge held within an LMS, is accumulated over time [39]. Accordingly, certain forms of knowledge may be prioritized in individual and collective memories as a function of the advantages that they offer. Several studies have suggested that only certain components of knowledge acquired individually are preferably stored and transferred because they offer adaptive advantages [40,41]. In this case, experts from the communities prioritized the acquisition of knowledge of common illnesses over knowledge of severe illnesses.

### Sharing the knowledge of treatment

Few experts share knowledge of the treatment of therapeutic targets, making the system vulnerable to the disappearance of information on medicinal plants. These results agree with the assumptions in the literature, which has long sought to study patterns of information sharing within medical systems [42,43].

In analyzing sharing processes from two perspectives, it was found that there is a greater degree of consensus on knowledge of therapeutic targets than of medicinal plants that are used to treat them (the sharing of information units). These results show that while knowledge of certain illnesses may be widespread among experts, they know different treatments for each one, i.e., the redundancy levels found for certain therapeutic targets are divided among the informants, indicating that information is not being shared among experts. In this case, it can be hypothesized that LMSs are built from knowledge acquired through the experience of individual experts.

This study did not aim to examine the factors that have caused high or low degrees of knowledge sharing; we merely assumed that if high degrees of sharing occur among experts, the system is more protected from information loss and is thus resilient. However, we recognize the importance of this question and will discuss possible factors that may have led to low degrees of knowledge transmission between experts.

Low degrees of consensus among experts may be attributable to the varying roles that experts play in different areas of the medical system. For example, some experts may be responsible for providing knowledge of spiritual diseases, while others may focus on children's diseases, leading each to specialize in a specific field. Alternatively, experts may prefer to keep their knowledge secret, guaranteeing their reputation as a therapeutic expert and thereby limiting the transmission of knowledge [44]. However, neither of these possibilities appears to be present in the communities studied given that, according to interviews and direct observations, many experts maintain amicable relations with other experts and are thus interested in sharing information with their peers.

It may also be possible that apparently idiosyncratic information held by an expert may actually be well disseminated among non-experts. Knowledge sharing among local experts may be less prevalent than knowledge sharing between other community members. Boster and Johnson [45], for example, found higher degrees of consensus among lay people than among experts when comparing degrees of information sharing on tasks related to fish among fishermen and among other individuals with no fishing experience. Focusing on the use of medicinal plants, Vandebroek [44] also found that people of two communities in Bolivia who know more about medicinal plant were less likely to share information on this subject with others.

Given that experts are the major producers of knowledge, while others copy information produced by them, it is interesting to identify what information is most widespread throughout a community. Lay people may follow trends when copying, and they do not necessarily take up random information from any person [46]. This biased transmission of knowledge may be based on an expert’s level of prestige or on the adaptive importance of information to a community. An analysis from this perspective may elucidate important facets of LMS resilience. For the communities examined in the present work, there do not appear to be major obstacles to information sharing among experts, and it would therefore be expected that if information was widespread among laymen, it would most likely reach other experts.

In the context of information sharing, another important perspective is the exchange of information between neighboring communities, as Berkes & Jolly [47] have previously noted in their work on the resilience of Inuit communities. Although the present work has not analyzed the communities from this perspective, the exchange of knowledge between the studied communities or between them and other communities did not seem to be very significant.

Thus, two other explanations appear to apply best to these findings: 1) as mentioned above, part of the system is still being constructed through each expert’s method of trial and error, and knowledge generated is thus only transmitted after being validated over time; 2) experts have different life histories, carrying with them knowledge that has been acquired from elsewhere, and given the amount of time that has passed since both communities were established, significant knowledge transmission between the communities may not yet be possible. The innovation of knowledge can be very costly, despite its importance for redundancy; thus, it seems that the second situation operates more strongly in the distribution of knowledge, although both can act together. From this perspective, the low degree of knowledge sharing may reflect the evolutionary nature of such systems. After information is validated by the community, it may become more widely known across the community.

### Limitations of this work

This study aims to investigate the resilience of local medical systems from the perspectives of local experts, and it was not based on community perspectives as a whole. We chose to study individuals in this sector because they are recognized as persons that ill individuals consult when illness occurs. This limitation prevents us from expanding the results to the entire population, which may display different medicinal plant-use behaviors. However, investigating resilience based on the sector that carries a significant proportion of the medicinal plant knowledge also reveals the major points of vulnerability within the larger health system because it is probable that limitations in this sector will cause other individuals to exhibit similar shortcomings when collecting information from experts.

As in many ethnobiological studies, the data collection for this study was based on informant perspectives, which can be influenced by several factors at the time of data collection. This study focuses on medical systems that are constructed by social actors, who add taxa to the medicinal collection according to their perception of the illnesses and of the species. Thus, our priority was to use those data rather than any data from other sources, as those other data could not reflect the characteristics of the local system but would represent other medical systems. Accordingly, the methods used here were devised following intense analyses of the characteristics of LMSs studied through direct observation, thereby avoiding possible biases. Quantitative data were utilized to investigate resilience, but we emphasize that knowledge about the studied community can help one to choose the methodology as well as to relativize the results.

The communities studied have a small population and live amid similar specific conditions. For this reason, we tried to discuss their main features to contextualize the LMSs. This specificity does not invalidate the findings but limits the range of potential generalization, as in the case of the use of redundant species in the absence of preferred species. However, the main findings of this study can be reflected in any community, rural or urban, and we discussed different situations that can influence the resilience of LMSs according to our vision of the URM.

## Conclusions

In analyzing the LMSs of two communities, we did not aim to measure their resilience because resilience has influences with various origins and because these influences interact. As was stated above, we wanted to clarify specific features that make an LMS more or less vulnerable to certain disturbances, especially those that make species and local knowledge unavailable. Despite the low redundancy found in the studied LMSs, redundant species have been shown to be crucial in the absence of preferred species. The expert informants demonstrated that they focus on treatments for more frequent illnesses, focusing on higher-redundancy species in treating recurring medical events. In contrast, severe illnesses appear to be more vulnerable to disturbances. In terms of the URM, this study call into question the role of redundancy in LMS resilience if no sharing of knowledge occurs between people. Therefore, we suggest that knowledge sharing must always be considered in studies of resilience.

According to the answers of the hypotheses obtained, we determined that important factors are acting together. In the systems examined, there are features that contribute to the resilience of an LMS, whereas other features may represent an obstacle to the functioning of the system. How strongly each feature can act to influence resilience depends on the nature of the disturbance that can occur. We intend to discuss other questions that may be related to the results we obtained and hope that future studies will consider these aspects to allow a deeper analysis of the resilience of local medical systems, considering the importance of such resilience to the permanence of local communities and their traditional ecological knowledge.

## Supporting Information

S1 Table Cited therapeutic targets and their redundancy, frequency, perceived severity and medical system sharing levels for two rural communities in the semi-arid region of northeastern Brazil. (DOCX)

S2 Table Description of cited therapeutic targets or their equivalent condition in Western medicine. (DOCX)
