# Supplementary material for: Does Plant Species Richness Guarantee the Resilience of Local Medical Systems? A Perspective from Utilitarian Redundancy
Source: PLoS One. 2015 Mar 20;10(3):e0119826. doi: 10.1371/journal.pone.0119826 (PMC4368708; doi:10.1371/journal.pone.0119826)
Supplement: S1 Table — (DOCX) [file pone.0119826.s001.docx]

**S1 Table: Cited therapeutic targets and their redundancy, frequency, perceived severity and medical system sharing levels for two rural communities in the semi-arid region of northeastern Brazil.**

| **Assentamento 10 de Abril** | **Sítio Bréa** |
| --- | --- |

| Therapeutic Target | No. of plants | Redundancy level | Freq. | Sharing | Body system | Therapeutic Target | No. of plants | Redundancy level | Freq. | Sharing | | Body system | |
| --- | --- | --- | --- | --- | --- | --- | --- | --- | --- | --- | --- | --- | --- |
| Cold ² | 50 | HR | 10 | 19 | RS | Cold ² | 34 | HR | 10 | 22 | | RS | |
| Headache ² | 19 | HR | 10 | 13 | NC | Fever ¹ ² | 24 | HR | 10 | 16 | | NC | |
| Fever ¹ ² | 19 | HR | 9 | 13 | NC | Bronchitis ¹ | 19 | HR | 1 | 9 | | RS | |
| Coughing ² | 17 | R | 10 | 3 | RS | Inflammation | 18 | HR | 10 | 4 | | NC | |
| Food that offends ² | 15 | R | 9 | 6 | DS | Blood pressure ¹ | 17 | R | 8 | 12 | | HS | |
| Bronchitis ¹ | 15 | R | 6 | 13 | RS | Cut ² | 16 | R | 9 | 18 | | IN | |
| Uterine inflammation¹ ² | 14 | R | 9 | 14 | GUS | Headache ¹ ² | 16 | R | 9 | 13 | | NC | |
| Bellyache ² | 11 | R | 10 | 7 | DS | Uterine inflammation ¹ | 15 | R | 1 | 13 | | GUS | |
| Blow ² | 11 | R | 10 | 10 | IN | Food that offends ¹ ² | 14 | R | 8 | 15 | | DS | |
| Colic ² | 10 | R | 9 | 7 | GUS | Sinusitis | 13 | R | 7 | 7 | | RS | |
| Postpartum rest | 10 | R | 6 | 6 | PBP | Throat ² | 12 | R | _ | 7 | | RS | |
| Blood pressure ¹ | 9 | R | 10 | 8 | SC | Lung | 11 | R | 10 | 5 | | RS | |
| Thick blood | 9 | R | 7 | 1 | HS | Blow ² | 10 | R | 10 | 15 | | IN | |
| Kidney pain | 9 | R | 6 | 10 | GUS | Dysentery | 10 | R | 8 | 4 | | DS | |
| Intestine | 9 | R | 2 | 3 | DS | Gastritis ² | 10 | R | 8 | 6 | | DS | |
| Toothache | 8 | R | 8 | 4 | MSS | Cough ² | 9 | R | 10 | 8 | | RS | |
| Vermin | 8 | R | 8 | 7 | IPD | Stomach ache ¹ ² | 9 | R | 10 | 13 | | DS | |
| Tooth eruption² | 7 | R | 10 | 10 | ED | Insomnia | 9 | R | 6 | 4 | | SN | |
| Blood flow | 7 | R | 9 | 7 | SC | Thick blood ² | 9 | R | 2 | 5 | HS | |  |
| Diabetes ¹ | 7 | R | 8 | 8 | ED | Vermin ¹ ² | 8 | R | 8 | 10 | | IPD | |
| Cancer ¹ | 7 | R | 4 | 7 | NEO | Heart ¹ | 8 | R | 2 | 3 | | SC | |
| Gastritis ² | 6 | R | 9 | 1 | DS | Stroke ¹ | 8 | R | 0 | 3 | | SN | |
| Liver | 6 | R | 5 | 3 | DS | Pneumonia ¹ | 6 | R | 9 | 5 | | RS | |
| Anemia ¹ ² | 6 | R | 4 | 2 | HS | Fatigue | 6 | R | 9 | 2 | | RS | |
| Angry wound ¹ | 6 | R | 0 | 6 | IN | Ennui | 6 | R | 4 | 6 | | DS | |
| Joint pain | 6 | R | _ | 6 | MSS | Tooth eruption² | 6 | R | 1 | 4 | | ED | |
| Heartburn ² | 5 | LR | 10 | 4 | DS | Gastric ulcer | 6 | R | _ | 3 | | DS | |
| Sore throat ¹ | 5 | LR | 8 | 6 | RS | Nail head ² | 5 | LR | 10 | 5 | | SD | |
| Sinusitis | 5 | LR | 8 | 6 | RS | Back pain | 5 | LR | 9 | 4 | | MSS | |
| Nail head | 5 | LR | 7 | 2 | SD | Toothache ² | 5 | LR | 9 | 4 | | MSS | |
| Kidney stone | 5 | LR | 7 | 4 | GUS | Wound | 5 | LR | 3 | 4 | | IN | |
| Stroke ¹ | 5 | LR | 3 | 3 | SN | Uterine wound | 5 | LR | 3 | 1 | | GUS | |
| Ramo | 5 | LR | 1 | 8 | EAD | Heartburn | 5 | LR | 3 | 6 | | DS | |
| Burn ² | 4 | LR | 10 | 6 | IN | Constipation | 5 | LR | 2 | 3 | | DS | |
| Heart ¹ | 4 | LR | 4 | 3 | SC | Prostate | 5 | LR | _ | 1 | | GUS | |
| Osteoporosis | 4 | LR | 1 | 3 | MSS | Burn | 5 | LR | _ | 3 | | IN | |
| Back pain ² | 3 | LR | 10 | 3 | MSS | Ramo ¹ ² | 4 | LR | 9 | 3 | | EAD | |
| Stress | 3 | LR | 10 | 3 | SN | Anemia ¹ | 4 | LR | 8 | 4 | | HS | |
| Earache | 3 | LR | 5 | 4 | EMD | Asthma ¹ | 4 | LR | 6 | 5 | | RS | |
| Blood cramp ¹ | 3 | LR | 3 | 3 | DS | Colic ² | 4 | LR | 4 | 5 | | GUS | |
| Unheiro | 3 | LR | 2 | 1 | SD | Malnutrition | 4 | LR | 3 | 1 | | ED | |
| Constipation | 2 | LR | 7 | 2 | DS | Diabetes ¹ | 4 | LR | 3 | 5 | | ED | |
| Labyrinthitis | 2 | LR | 7 | 1 | EMD | Vaginal inflammation | 4 | LR | 2 | 2 | | GUS | |
| Depression ¹ | 2 | LR | 5 | 3 | SN | Kidney | 4 | LR | _ | 4 | | GUS | |
| Gastric ulcer ¹ | 2 | LR | 4 | 3 | DS | Blood cramp ¹ | 3 | LR | _ | 3 | | DS | |
| Prostate ¹ | 2 | LR | 3 | 1 | GUS | Cancer ¹ | 3 | LR | 10 | 4 | | NEO | |
| Bone fracture ¹ | 2 | LR | 3 | 3 | IN | Itch | 3 | LR | 8 | 3 | | SD | |
| Miscarriage | 2 | LR | 2 | 2 | PBP | Child colic | 3 | LR | 8 | 2 | | DS | |
| Itch ² | 2 | LR | 1 | 1 | SD | Estalicido | 3 | LR | 7 | 1 | | RS | |
| Mouth wound | 2 | LR | 1 | 3 | IPD | Stress | 3 | LR | 2 | 2 | | NS | |
| Pneumonia ¹ | 2 | LR | 1 | 1 | RS | Liver | 3 | LR | 2 | 2 | | DS | |
| Measles ¹ ² | 2 | LR | 0 | 2 | IPD | Intestine | 3 | LR | 1 | 4 | | DS | |
| Fatigue | 2 | LR | _ | 6 | RS | Bone fracture | 3 | LR | _ | 3 | | IN | |
| Mind | 2 | LR | _ | 1 | NS | Congestion ¹ | 2 | LR | 10 | 1 | | DS | |
| Cholesterol ¹ | 1 | LR (NR) | 10 | 2 | ED | Whooping cough | 2 | LR | 9 | 1 | | RS | |
| Allergy | 1 | LR (NR) | 9 | 1 | RS | Earache | 2 | LR | 5 | 1 | | EMD | |
| Menstrual problem | 1 | LR (NR) | 9 | 1 | ED | Epilepsy | 2 | LR | 3 | 1 | | NS | |
| Ennui ² | 1 | LR (NR) | 6 | 3 | DS | Hemorrhoids | 2 | LR | 1 | 1 | | IPD | |
| Asthma ¹ | 1 | LR (NR) | 5 | 1 | RS | Skin ulcer | 2 | LR | 1 | 1 | | SD | |
| Mycosis | 1 | LR (NR) | 4 | 1 | SD | Rheumatism | 2 | LR | 1 | 1 | | NS | |
| Childbirth pain | 1 | LR (NR) | 3 | 1 | PBP | Giddiness | 2 | LR | 0 | 1 | | EMD | |
| Conjunctivitis | 1 | LR (NR) | 1 | 1 | EAD | Allergy ² | 1 | LR (NR) | 1 | 2 | | RS | |
| Vein obstruction | 1 | LR (NR) | 1 | 5 | SC | Blister ¹ | 1 | LR (NR) | _ | 1 | | IPD | |
| Tuberculosis ¹ | 1 | LR (NR) | 1 | 1 | RS | Cholesterol ¹ | 1 | LR (NR) | 9 | 1 | | ED | |
| Blister | 1 | LR (NR) | 0 | 1 | IPD | Postpartum pain | 1 | LR (NR) | 8 | 3 | | PBP | |
| Chickenpox ¹ | 1 | LR (NR) | 0 | 1 | IPD | Thorn | 1 | LR (NR) | 8 | 1 | | IN | |
| Mumps ¹ | 1 | LR (NR) | 0 | 1 | IPD | Angry wound | 1 | LR (NR) | 7 | 1 | | IN | |
| Chilblain | 1 | LR (NR) | _ | 2 | IPD | Swelling | 1 | LR (NR) | 6 | 1 | | DS | |
| Vaginal inflammation | 1 | LR (NR) | _ | 1 | GUS | Dry intestine | 1 | LR (NR) | 3 | 1 | | DS | |
| Insomnia | 1 | LR (NR) | _ | 1 | NS | Menopause | 1 | LR (NR) | 3 | 1 | | ED | |
| Hair loss | 1 | LR (NR) | _ | 1 | SD | Osteoporosis | 1 | LR (NR) | 2 | 1 | | MSS | |
| Heat (quentura) | 1 | LR (NR) | _ | 3 | DS | Mumps ² | 1 | LR (NR) | 2 | 1 | | IPD | |
| Dengue ¹ | 0 | LR (NT) | _ | 0 | IPD | Childbirth | 1 | LR (NR) | 1 | 1 | | PBP | |
| Arthritis | 0 | LR (NT) | _ | 0 | MSS | Pasty eye | 1 | LR (NR) | 1 | 1 | | EAD | |
| Arthrosis | 0 | LR (NT) | _ | 0 | MSS | Liver stone | 1 | LR (NR) | 0 | 1 | | DS | |
| Blindness | 0 | LR (NT) | _ | 0 | EAD | Measles ¹ | 1 | LR (NR) | _ | 1 | | IPD | |
| Leukemia ¹ | 0 | LR (NT) | _ | 0 | NEO | Jaundice | 1 | LR (NR) | _ | 1 | | IPD | |
| Glaucoma | 0 | LR (NT) | _ | 0 | EAD | Vilida | 1 | LR (NR) | _ | 1 | | EAD | |
| AIDS¹ | 0 | LR (NT) | _ | 0 | HS | Water belly ¹ | 0 | LR (NT) | _ | 0 | | IPD | |
| Chirrosis¹ | 0 | LR (NT) | _ | 0 | DS | Chickenpox | 0 | LR (NT) | _ | 0 | | IPD | |
| Meningitis ¹ | 0 | LR (NT) | _ | 0 | IPD | Leishmaniasis | 0 | LR (NT) | _ | 0 | | IPD | |
| Hepatitis ¹ | 0 | LR (NT) | _ | 0 | DS | Tuberculosis ¹ | 0 | LR (NT) | _ | 0 | | RS | |
| Hansen’s disease ¹ | 0 | LR (NT) | _ | 0 | IPD | Tumor ¹ | 0 | LR (NT) | _ | 0 | | NEO | |
| Herniated disk ¹ | 0 | LR (NT) | _ | 0 | MSS | AIDS ¹ | 0 | LR (NT) | _ | 0 | | HS | |
| Leishmaniasis ¹ | 0 | LR (NT) | _ | 0 | IPD | Chagas disease ¹ | 0 | LR (NT) | _ | 0 | | IPD | |
| Appendicitis ¹ | 0 | LR (NT) | _ | 0 | DS | Cirrhosis ¹ | 0 | LR (NT) | _ | 0 | | DS | |
| Cholera ¹ | 0 | LR (NT) | _ | 0 | IPD | Tetanus ¹ | 0 | LR (NT) | _ | 0 | | IPD | |
| Chagas disease ¹ | 0 | LR (NT) | _ | 0 | IPD | Leukemia ¹ | 0 | LR (NT) | _ | 0 | | NEO | |
| Tiriça ¹ | 0 | LR (NT) | _ | 0 | IPD | Yellow fever ¹ | 0 | LR (NT) | _ | 0 | | IPD | |
| Gallbladder ¹ | 0 | LR (NT) | _ | 0 | DS | Cholera ¹ | 0 | LR (NT) | _ | 0 | | IPD | |

Species redundancy: HR - highly redundant; R - Redundant; LR - less redundant; NR - no redundancy; NT - no treatment. Perceived severity: ¹ - therapeutic targets cited among the most severe targets; ² - therapeutic targets cited among the least severe targets. Frequency of occurrence (freq.): number assigned during ranking in the participatory workshop, ranging from 10 - most frequent to 1 - least frequent and 0 - no occurrence. Therapeutic targets marked with “_” were not ranked due to a lack of consensus realized in the workshop. Therapeutic target sharing (sharing): number of experts who cited any treatment for the therapeutic target. Body systems according to the WHO (2007): DS - Diseases of the digestive system, RS - Diseases of respiratory system, GUS - Diseases of genitourinary system, IN - Injury, poisoning and certain other consequences of external causes , MSS - Diseases of musculoskeletal system and connective tissue, ED - endocrine, nutritional and metabolic diseases, SD - diseases of skin and subcutaneous tissue, HS - Diseases of the blood and blood-forming organs and certain disorders involving the immune mechanism, CS - Diseases of the circulatory system, NS - diseases of the nervous system, IPD - infectious and parasitic diseases, PBP - pregnancy, childbirth and puerperium, EMD - Diseases of ear and mastoid process, NEO - neoplasm, EAD - diseases of eye and adnexa, NC - non-classified symptom.
