# Supplementary material for: Does Plant Species Richness Guarantee the Resilience of Local Medical Systems? A Perspective from Utilitarian Redundancy
Source: PLoS One. 2015 Mar 20;10(3):e0119826. doi: 10.1371/journal.pone.0119826 (PMC4368708; doi:10.1371/journal.pone.0119826)
Supplement: S2 Table — (DOCX) [file pone.0119826.s002.docx]

**S2 Table.** **Description of cited therapeutic targets or their equivalent condition in Western medicine.**

| Therapeutic Target | Description of therapeutic target or equivalent condition in Western medicine |
| --- | --- |
| AIDS | AIDS |
| Allergy | Respiratory allergy |
| Anemia | Anemia |
| Angry wound | "A wound that takes longer than one month to heal" |
| Appendicitis | Appendicitis |
| Arthritis | Arthritis |
| Arthrosis | Arthrosis |
| Asthma | Asthma |
| Back pain | Any back pain that can be caused by fatigue or poor posture |
| Bellyache | Any bellyache of unknown cause |
| Blindness | Blindness |
| Blister | Smallpox |
| Blood cramp | An illness that results in bloody diarrhea |
| Blood flow | Poor circulation that can cause varicose veins |
| Blood pressure | High blood pressure |
| Blow | Hematomas caused by blows |
| Bone fracture | Bone fracture |
| Bronchitis | Bronchitis |
| Burning pain | Skin burn |
| Cancer | “Goiter, irritated skin, hair loss” (Ass) or "a lumb anywhere in the body" (Br) |
| Chagas disease | American trypanosomiasis |
| Chickenpox | Varicella |
| Chilblain | An infection in the toes caused by fungi |
| Child colic | Infantile colic |
| Childbirth pain | Labor pain |
| CirrHosis | CirrHosis |
| Cholera | Cholera |
| Cholesterol | High cholesterol |
| Cold | Influenza |
| Colic | Menstrual pain |
| Congestion | Chronic heart failure |
| Conjunctivitis | Conjunctivitis |
| Constipation | Costiveness or dyschezia |
| Coughing | Any cough of unknown cause |
| Cut | Superficial cut in the skin |
| Dengue | Dengue fever |
| Depression | Depression |
| Diabetes | Diabetes mellitus |
| Dry intestine | A condition that can cause constipation |
| Dysentery | Dysentery |
| Earache | Ear infection |
| Ennui | Lack of appetite |
| Epilepsy | Epilepsy |
| Estalicido | An allergy originated as a “problem of the face” that is not chronic |
| Fatigue | A chronic respiratory disease in which phlegm accumulates as fever develops |
| Fever | Any fever of unknown cause |
| Food that offends | "Instances when food causes indigestion" |
| Gallbladder | Problems caused by the presence of gallstones |
| Gastric ulcer | Gastric ulcer |
| Gastritis | Gastritis |
| Giddiness | A recurring state of dizziness that differs from labyrinthitis |
| Glaucoma | Glaucoma |
| Hair loss | "Can be caused by cancer" |
| Hansen’s disease | Hansen’s disease |
| Headache | Any headache of unknown cause |
| Heart | Heart attack |
| Burning pain | Burning Sensation in Stomach |
| Heat (quentura) | A feeling of heat throughout the body |
| Hemorrhoids | Oxyuriasis |
| Hepatitis | Hepatitis |
| Herniated disk | Herniated disk |
| Inflammation | Internal or external inflammation |
| Insomnia | Insomnia |
| Intestine | Inflammation in the intestine that causes gases |
| Itch | Scabies |
| Jaundice | Icterus |
| Joint pain | Painful joints |
| Kidney pain | Pain in the lumbar attributed to kidney inflammation |
| Kidney stone | Renal calculus |
| Labyrinthitis | Labyrinthitis |
| Leishmaniasis | Leishmaniasis |
| Leukemia | An illness “that turns blood into water", it is not the same as cancer |
| Liver | Unidentified liver inflammation, which is distinct from hepatitis and cirrhosis |
| Lung | Lungs infection that can cause pneumonia |
| Malnutrition | Malnutrition |
| Measles | Measles |
| Meningitis | Meningitis |
| Menopause | Menopause |
| Menstrual problem | Unregulated menstruation |
| Mind | Any mental problems |
| Miscarriage | Artificial death of an embryo or fetus in the womb |
| Mouth wound | Stomatitis |
| Mumps | Mumps |
| Mycosis | A fungal infection on the skin |
| Nail head | A type of inflammation that involves the formation of pus on the skin |
| Osteoporosis | Osteoporosis |
| Pasty eye | the presence of thick mucus in the eyes |
| Pneumonia | Pneumonia |
| Postpartum rest | Postpartum rest |
| Prostate | Prostate inflammation |
| Ramo | An eye problem that “hinders sight after a wind passes" |
| Rheumatism | Rheumatism |
| Sinusitis | Sinusitis |
| Skin ulcer | Skin ulcer |
| Sore throat | Sore throat |
| Stomach ache | Any stomach ache of unkown cause |
| Stress | Stress |
| Stroke | Cerebrovascular accident |
| Swelling | Fluid retention in the body |
| Tetanus | Tetanus |
| Thick blood | A blood irregularity that results in thrombosis and reduced immunity |
| Thorn | A thorn of a plant on skin |
| Throat | Hoarseness |
| Tooth eruption | Emergence of various diseases related to tooth birth period in babies |
| Toothache | Odontalgia |
| Tuberculosis | Tuberculosis |
| Tumor | "A type of inflammation in na part of the body" |
| Unheiro | Paronychia |
| Uterine inflammation | Uterine inflammation |
| Vaginal inflammation | Urinary infection |
| Vein obstruction | Vein obstruction |
| Vermin | Any type of verminosis |
| Vilida | “A burning sensation in the eyes that causes poor eyesight.” |
| Water belly | Schistosomiasis |
| Whooping cough | Pertussis |
| Wound | Wound |
| Yellow fever | Yellow fever |
